# An Automated Centrifugal Microfluidic Platform for Efficient Multistep Blood Sample Preparation and Clean-Up towards Small Ion-Molecule Analysis

**DOI:** 10.3390/mi14122257

**Published:** 2023-12-18

**Authors:** Yuting Hou, Rohit Mishra, Yufeng Zhao, Jens Ducrée, Jed D. Harrison

**Affiliations:** 1 Department of Chemistry, University of Alberta, Edmonton, AB T6G 2G2, Canada; yufeng@ualberta.ca (Y.Z.); jed.harrison@ualberta.ca (J.D.H.); 2 FPC@DCU—Fraunhofer Project Centre for Embedded Bioanalytical Systems, Dublin City University, D09 V209 Dublin, Ireland; 3 School of Physical Sciences, Dublin City University, D09 V209 Dublin, Ireland; jens.ducree@dcu.ie; 4 Centre for Research and Applications in Fluidic Technologies, National Research Council Canada, Toronto, ON M5S 3G8, Canada; 5 Leslie Dan Faculty of Pharmacy, University of Toronto, Toronto, ON M5S 3M2, Canada

**Keywords:** whole blood sample preparation, microfluidics, lab-on-a-disc, small molecule analysis, mass spectrometry

## Abstract

Sample preparation for mass spectroscopy typically involves several liquid and solid phase clean-ups, extractions, and other unit operations, which are labour-intensive and error-prone. We demonstrate a centrifugal microfluidic platform that automates the whole blood sample’s preparation and clean-up by combining traditional liquid-phase and multiple solid-phase extractions for applications in mass spectroscopy (MS)-based small molecule detection. Liquid phase extraction was performed using methanol to precipitate proteins in plasma separated from a blood sample under centrifugal force. The preloaded solid phase composed of C18 beads then removed lipids with a combination of silica particles, which further cleaned up any remaining proteins. We further integrated the application of this sample prep disc with matrix-assisted laser desorption/ionization (MALDI) MS by using glancing angle deposition films, which further cleaned up the processed sample by segregating the electrolyte background from the sample salts. Additionally, hydrophilic interaction liquid chromatography (HILIC) MS was employed for detecting targeted free amino acids. Therefore, several representative ionic metabolites, including several amino acids and organic acids from blood samples, were analysed by both MALDI-MS and HILIC-MS to demonstrate the performance of this sample preparation disc. The fully automated blood sample preparation procedure only took 35 mins, with a throughput of three parallel units.

## 1. Introduction

Blood is one of the most informative samples for bioanalysis [1], and it can accurately reflect the state and progression of many diseases [2]. Target molecules within blood samples are encompassed by various “omics”, including genomics, transcriptomic, proteomics, and metabolomics [3]. There is overwhelming evidence showing how metabolites function as signalling molecules to regulate gene expression, protein activities, and, most importantly, disease phenotype [4,5]. Recent studies in metabolomics clearly demonstrate that “the metabolites are perhaps the body’s most important signaling molecules” [6]. The roles and activity of metabolites offer fields of great potential that need to be fully researched. Blood ion metabolites have served as new biomarkers for major diseases such as chronic kidney disease, cancer, and neurodegenerative diseases. For example, the aspartic acid in peripheral blood is reported in the diagnostic panel for Alzheimer’s disease [7]. The taurine level in the blood has been demonstrated to be remarkably influenced in chronic kidney disease patients by a large-population investigation [8].

However, the biological sample matrix is extremely complex in the blood, and hence, the extraction of the target molecule from the biological sample, along with purification, is required for analysis. At the same time, most often, sample preparation procedures for blood analysis require skilled technicians and well-equipped, expensive laboratories. Microfluidics and related technologies are being explored to create automated, cost-effective, and rapid solutions for a wide variety of blood analyses. For example, Kitamori et al. combined the axial migration effect and filtration to obtain blood-cell-free plasma in a microfluidic chip [9]. And Dixon et al. developed a completely automated blood–plasma separation process on a digital microfluidic device [10]. However, since most blood-related microfluidic devices are developed only for blood–plasma separation [11], the chemical complexity of the sample matrix in plasma remains high, and hence, further processing is often necessary. 

Centrifugal microfluidics, also called lab-on-a-disc, is a branch of microfluidic technology that employs centrifugal force to achieve on-disc flow control. The centrifugal force allows for ease of flow control while handling several operations and fluids in parallel, and, hence, makes it easier to achieve a simple and compact instrument setting without the requirement of external pumps, as in most conventional microfluidic technologies [12]. Several functional valves have been developed and can be applied on the centrifugal platform to realize metering, mixing, and switching based on the centrifugal force [13]. Centrifugal microfluidic discs are able to perform multiple steps and are utilized in clinical chemistry, immunodiagnostics, cell handling, and molecular diagnostics, as well as in food, water, and soil analysis [12]. To facilitate the scale-up of such technologies, integrated systems and mass-manufacturing methods have also been implemented [14], thus allowing for high commercialization potential [15,16,17]. Most blood-related lab-on-a-disc devices are developed for the purpose of preparing cell-free plasma [18]. Blood sample preparation involving multi-phase multi-step extractions (for further protein clean-up and small molecule extraction) is still a challenging area. Herein, we present a centrifugal microfluidic disc platform that can complete blood sample preparation towards mass spectroscopy by using a purely rotational flow control mechanism, fully automating the unit operations of the concept developed in a previous work [19]. We demonstrate significant stability and extent of coverage of sample processing steps by fully integrating an end-to-end microfluidic sample preparation and reagent handling process. Additionally, the advanced platform presented herein exhibits desirable properties, including limited toughness to organic solvents in the process and stability at high rotational speeds. The design allows for a minimal instrumentation approach. Key steps to be achieved on the disc include protein precipitation and sample recovery. In other words, many processing steps that are manually performed still rely on external equipment, such as a vortex and vacuum chamber, which involve unreliable manual transfers. By utilizing advanced microfluidic fluid control and systems integration with a unique design, we realize a fully on-disc, automated, multi-step blood sample processing method, eliminating the requirement for any manual operation and any external equipment other than a simple spindle motor. An additional key feature here is the ability to immediately process a freshly taken blood sample, which would allow for efficient quenching of the metabolism and, hence, conquer the instability of biomarkers that occur during transport and storage [20,21]. 

Even though there is no single technique that can be used to evaluate the full metabolome present in bio-samples, nuclear magnetic resonance (NMR) spectroscopy and mass spectrometry (MS) are the two main platforms for polar metabolites [22,23,24]. Although NMR is a non-destructively quantitative approach, it comes with the major disadvantages of low sensitivity and spectral resolution that make it challenging to obtain accurate small ion–molecule metabolite identification and quantification [25]. Mass spectrometry (MS)-based methods, especially coupled with separation approaches such as gas chromatography (GC) and liquid chromatography (LC), are excellent approaches in terms of high sensitivity and specificity, high throughput, and high accuracy for small molecule detection [26]. Amino acids are essential nutrients that also play an important role in revealing the metabolic pathways of many major diseases, like Alzheimer’s and Parkinson’s, and early detection of these diseases as well as different types of cancers [27,28,29]. Thus, we chose to analyse unlabelled free amino acids obtained from a microfluidic-disc-processed sample by both LC-MS [30] and MALDI-MS [31] to verify the performance of our on-disc blood sample processing method. The goal was to create a device that we can provide for end-to-end automation, and to obtain a cleaned-up, fully processed sample for subsequent MS analysis. This also demonstrated the ability of microfluidic platforms to provide laboratory automation for sample preparation, given that most microfluidic platforms are typically developed for sample-to-answer testing in decentralized settings. We anticipate our strategy to have broader implications that could provide for better flexibility towards the commercial deployment of microfluidic technologies.

The processed sample has been proven to be suitable for both HILIC-MS and MALDI-MS analyses. According to the Agilent HILIC-MS protocol for the analysis of 16 underivatized amino acids, all 16 of these amino acids in the disc-processed blood sample were separated and analysed by positive mode HILIC-MS using a HILIC column. We previously reported a modified GLAD thin film for MALDI-MS that offers rapid detection of several amino acids and organic acids with a high tolerance to salts [32]. MALDI-MS is employed during C18 and silica particle usage optimization by observing the crystallization of sample spots on GLAD film and comparing the S/N of several amino acid peaks. Also, quick estimations of specific amino acids are performed by MALDI-MS involving spiked isotopes.

## 2. Materials and Methods

### 2.1. GLAD Film Preparation for MALDI-MS

The MALDI chips were prepared as described previously [32]. Briefly, vertical silicon nano-posts were deposited on a silicon wafer substrate by glancing angle deposition (GLAD), followed by oxidation in an air environment and surface derivatization with (1*H*, 1*H*, 2*H*, 2*H*-perfluorooctyl) dimethylchlorosilane (pFMe2SiCl, Gelest). A sample of 60 ul (1*H*, 1*H*, 2*H*, 2*H*-perfluorooctyl) dimethylchlorosilane was diluted with 5 mL methanol in a glass Petri dish. By soaking the film in the diluted solution for 30 min at an ambient temperature, the silanol groups on the surface could be covalently fluorinated. Then, the GLAD film was laid flat in a Petri dish for air-drying and stored overnight for polymerization at room temperature.

### 2.2. Design and Fabrication of the Centrifugal Disc

**Figure 1:**
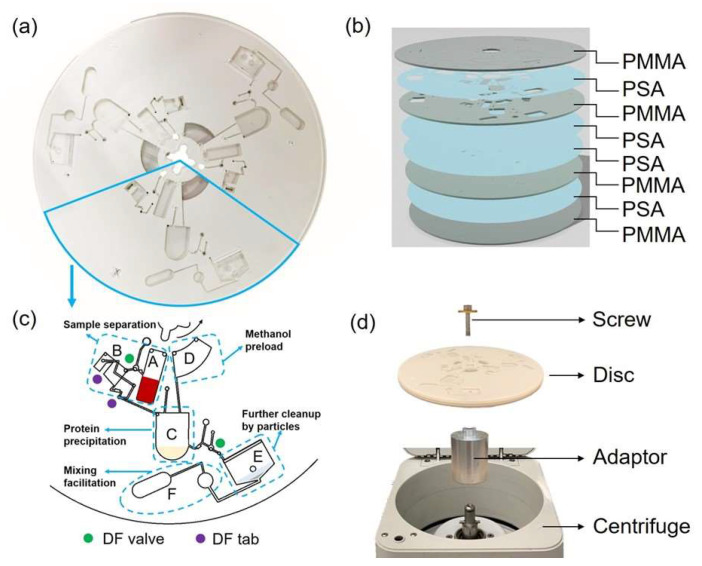
(**a**) Photograph of an assembled disc, which has three individual sample preparation units. (**b**) The order in which 4 layers of PMMA and 4 layers of PSA were stacked. The thickness of each PSA layer was 126 µm and the thickness of each PMMA layer was 1.5 mm. (**c**) Features of one individual unit from top perspective view. Design of top vent layer, microchannel layer, and lower channel layer are overlapped; top view of the overlap of all eight layers is given in Figure S1. Detailed designs are presented, including lower channels connecting reaction chambers. Coloured dots show locations where dissolvable film (DF) tabs were inserted. (**d**) Apparatuses employed to fix the disc to the centrifuge included the screw, which fit the adaptor; the disc; the custom-made adaptor; and the centrifuge without rotor (from top to bottom).

The disc was designed using computer-aided design software, AutoCAD 2018. Each disc (Փ = 15 cm) could accommodate three individual sample preparations, as seen in Figure 1a. Figure 1b shows the centrifugal microfluidic device consisting of four layers of poly (methyl methacrylate) (PMMA) (Acrylite FF, Johnston plastics, Edmonton, AB, Canada) 1.5 mm thick and four layers of pressure-sensitive adhesive (PSA) (ARcare 8393, Adhesives Research, Glen Rock, NJ, USA) 126 μm thick. Features were cut by CO_2_ laser ablation (Epilog Zing Laser Series, Golden, CO, USA), and aligned layers were laminated by a HL-100 rolling laminator laminator (Cheminstruments, Fairfield, CA, USA) with 100 psi, as shown in the order in Figure 1b. The PSA layer with adhesive on both sides served as the bonding layer to accomplish disc assembly while avoiding wet chemical procedures (Figure 1b) [33]. The dissolvable film valve was fabricated and inserted between the two adjacent PSA layers by following a previously described protocol [34]. Since all the PSA layers were opaque, the channel configuration on the lower PSA layer cannot be observed from the photo in Figure 1a. Therefore, the top view in AutoCAD is presented in Figure 1c to facilitate the visualization of the connection between the different reaction chambers. Further details and annotations of the design sketches are provided in the Supplementary Materials (Figure S1). The total cost of one disc is estimated to be less than fifteen dollars, and therefore, the cost of each parallel unit would be less than five dollars to accommodate the multistep processing procedures. The assembled disc was fixed on a centrifuge (Eppendorf 5415C centrifuge, Westbury, NY, USA) by a custom-made adaptor (Figure 1d).

### 2.3. Analysis of Processed Sample by Offline LC-MS

The separation of different amino acids was performed using a hydrophilic interaction chromatography column (InfinityLab Poroshell 120 HILIC-Z phase, Agilent Technologies, Santa Clara, CA, USA), while for detection, we used a Single Quadrupole MS (1100 HPLC with G1946A MSD, Agilent Technologies, Santa Clara, CA, USA) in positive ion mode. All reagents were HPLC grade or higher. Water was purified using an EMD Millipore Milli-Q Integral System (Darmstadt, Germany). For the separation of 16 free, underivatized amino acids, including phenylalanine (F), leucine (L), isoleucine (I), methionine (M), tyrosine (Y), valine (V), proline (P), alanine (A), threonine (T), glycine (G), serine (S), glutamic acid (E), aspartic acid (D), histidine (H), arginine (R), and lysine (K), we followed the method from Agilent to prepare the mobile phase and set instrument conditions [35]. Mobile phase A consisted of 20 mM ammonium formate in water at pH = 3.1, while mobile phase B consisted of 20 mM aqueous ammonium formate in 9:1 acetonitrile/water. The flow rate was 0.50 mL/min, with an injection volume of 0.2 µL, at a 30 °C column temperature.

### 2.4. Analysis of Processed Sample by MALDI-MS

A 35 μL sample was pipetted out of a disc after sample clean-up, then acidified with 2 M HCl for a final concentration of 0.18 M HCl. A 1.3 μL sample was then spotted onto a GLAD chip in a Petri dish and dried at 4 °C for salt crystallization. All chemicals, including amino acids, organic acids, and isotope standards, were purchased from Sigma and were prepared as a high-concentration stock solution in DI water. A customized MALDI plate was made to fit the MS inlet, adapting it to the thickness of the GLAD chip. Double-sided conductive carbon tape (Electron Microscopy Sciences, Hatfield, PA, USA) attached the GLAD film, with dried sample spots, to the MALDI plate. The MALDI plate was inserted into an AB Sciex Voyager Elite MALDI-TOF mass spectrometer for analysis. The nitrogen laser (337 nm, 3 ns pulse) pulse provided energy for both desorption and ionization. The carboxyl groups in AAs and organic acids could be easily ionized under negative ion mode. The mass spectrum reflected averaged ion counts for 100 laser shots while moving the beam to new locations in the spot. Each data point stood for the average value of an ion count or ion count-to-noise ratio of 3 replicate spots. Standard deviation was calculated the same way as for the sample preparation assay’s development and quantification. The laser intensity was set to 1850 (a.u.) for C18 and silica particle optimization; 2100 (a.u.) was used for the real sample measurements. All other information about the instrumental settings is listed in our previous work [32]. For rapid quantitative estimation, fresh blood samples were spiked with isotope standard stock solutions before the blood sample was loaded to the disc, followed by MS detection after on-disc clean-up. Ion counts were read from the MS spectrum by inputting a certain *m*/*z* value into the data analysis software Data Explorer 4.0.

## 3. Results

### 3.1. Workflow of On-Disc Blood Sample Preparation and Optimization

**Figure 2:**
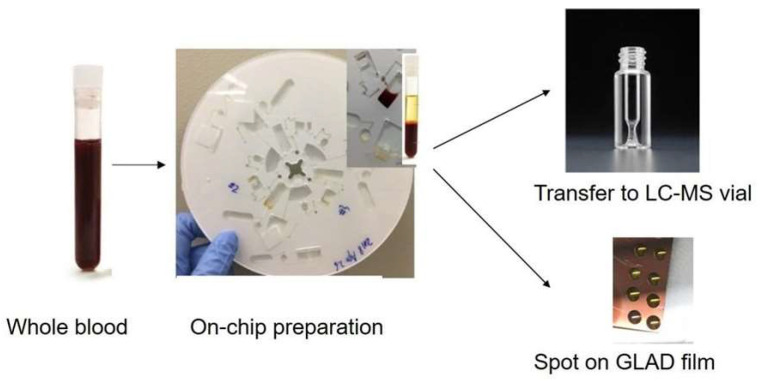
Workflow of on-chip sample preparation, followed by LC-MS and MALDI-MS. The operator loaded the sample of whole blood and all the reagents onto the microfluidic platform. All the operations needed for sample processing were performed on the disc using a standard centrifuge. The samples were thereafter transferred to input vials for LC-MS and GLAD film substrate for MALDI-MS.

Figure 2 illustrates the intended workflow for sample preparation using the lab-on-a-disc device. The goal was to introduce a whole blood sample; perform all processing steps within the device without further manual intervention; and then extract a 35 µL sample for the instrumental analysis of small, polar metabolites. In this study, the two methods employed for analysis were MALDI-MS, utilizing porous silicon films fabricated using the glancing angle deposition (GLAD) process, and HILIC-MS [36].

**Figure 3:**
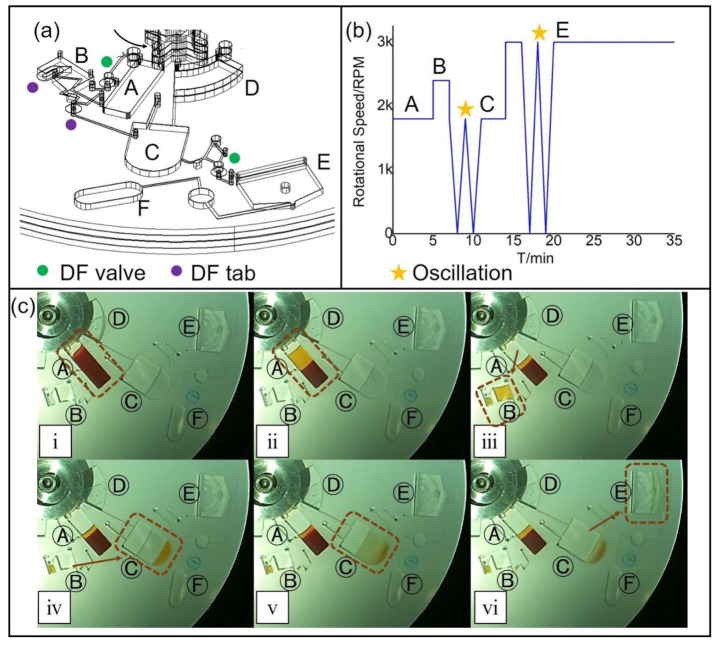
(**a**) 3D view and design for a single processing unit. The on-disc DF valves only burst at a specific spinning speed, while DF tabs open once wetted. (**b**) The spinning protocol. The oscillation part (W shaped area) is only for schematic purposes and depicts rapid shake-mode mixing previously demonstrated on several centrifugal microfluidic systems. (**c**) Image series of on-disc procedures: (**i**) Methanol is preloaded to chamber C, and then the blood sample is loaded to chamber A. (**ii**) Separation of whole blood takes place in chamber A at a constant spin speed (2400 rpm). (**iii**) Increased spin speed opens the DF valve and the plasma is transferred to metering chamber B. (**iv**) The surplus plasma in the small side chamber trigger dampens the DF tab and releases the metered 50 μL plasma to chamber C. (**v**) The mixing of plasma and methanol is completed by the oscillation of the spin speed between 0~2400 rpm. (**vi**) Increased spin speed opens the DF valve, and the supernatant after protein precipitation is transferred to chamber E.

We have previously established a sample clean-up procedure that could be used on a disc. However, the unit operations in that work were predominantly manual (unit operations such as blood separation, plasma metering, methanol, and C18 bead mixing for precipitating protein and removing lipids, filtration, mixing, phase separation with silica, and reconstitution for elution, among others) [19]. Fully integrating and automating all of the sample preparation steps on the disc, starting from whole blood, necessitated the need for the platform to not only integrate all the unit operations in a single end-to-end workflow, but also develop a system that can withstand the reagents. Hence, the workflow was optimized for a microfluidic platform with revised procedures, as discussed below in conjunction with Figure 3 and Video S1.

Figure 3 illustrates the various chambers on the disc, used to stage the sequential processing of a blood sample for ultimate delivery to a MALDI-MS surface or a HILIC-MS assay. The timing and rotational speeds in RPM, are shown in Table 1. Integrated dissolvable film (DF) burst valves were used to control the timing and release stage at each processing step. A 190 µL blood sample, collected from a healthy female volunteer in an ethylenediaminetetraacetic acid (EDTA)-coated tube, was loaded into plasma preparation chamber A. A suitable spin time to separate the cells from the plasma was easily determined by running the experiment under a video camera [37]. After 5 min of on-chip blood cell separation in chamber A at 1800 rpm, 50 μL of separated plasma was metered by chamber B, using the first DF-burst valve at 2400 rpm. The decanting DF valve was designed to be located much higher than the 45% volume line of the blood loading chamber to avoid transferring any blood cells after the separation [38].

The metered plasma was then diluted 3:1 (*v*/*v*) with preloaded methanol (LC/MS grade, Fisher Chemical, Ottawa, ON, Canada) in reservoir C for protein precipitation. The spinning speed was oscillated between 0 to 1800 rpm for 3 min to achieve thorough mixing below the DF valve burst frequency. Spinning the disc for 3 min at 1800 rpm then precipitated the aggregated proteins to the bottom of the chamber so that a filtration process was not required [19]. The supernatant was thereafter transferred to chamber E by increasing the spinning speed to the DF valve burst frequency of 3000 rpm. The removal of the remaining lipid and protein contaminants took place in the next step, by mixing with 10 μm C18 beads (S03207B, Silicycle, Quebec City, QC, Canada) and 235 nm silica nanoparticles (microParticles GmbH, Berlin, Germany) preloaded in chamber E. The optimum dose of C18 beads and silica nanoparticles was determined by comparing the signal-to-noise (S/N) value in the MS spectrum, as described below. Repeating the 3-min oscillating spinning speeds between 0 and 3000 rpm helped to achieve rapid mixing. Methanol was then rapidly evaporated away by spinning constantly at 3000 rpm for 15 min. At the same time, all particles were sedimented to the bottom of the reaction chamber in this step by centrifugal force. The methanol had to be removed to increase the surface tension of the supernatant so that small spots could form on the GLAD film. Thereafter, the supernatant was ready to be pipetted out for subsequent analysis.

### 3.2. Sample Preparation Assay Optimization for Blood Sample Preparation

Methanol precipitation was used as the primary method of protein removal from the metered plasma delivered by chamber A on the disc [39]. Lipid interferences were again removed with C18-coated silica particles, and any remaining proteins were removed with bare silica particles. These particles were preloaded into chamber E. They were then co-mixed with the methanol-treated plasma, and both particle types were pelleted by on-disc centrifugation.

**Figure 4:**
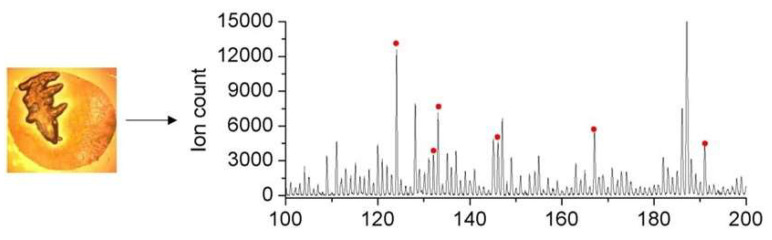
Mass spectrum for on-chip-processed blood sample spot; *m*/*z* peaks of the six analytes are labelled (taurine: 124.0, aspartic acid: 132.0, malic acid: 133.0, glutamic acid: 146.0, uric acid: 167.0, citric acid: 191.0). The desalting function of the GLAD film is illustrated by the crystallization of salts in the sample spot photo (left). S/N of these six analytes were employed to optimize the usage of C18 beads and silica particles.

Different amounts of C18-coated silica particles and bare silica particles were added to determine the optimum dose. The signal-to-noise ratios (S/N) of six amino acids and organic acids from the MALDI-MS spectrum were compared, and the conditions that gave the best SNR were selected. Figure 4 shows a typical MALDI-MS spectrum. The peaks of the six analytes are labelled by red dots in the MALDI-MS spectrum. Compared with the spectrum of the manually processed sample (Figure S2), no substantial signal loss was observed for our target ionic metabolites in the on-chip-prepared sample, demonstrating the significantly improved efficacy of the centrifugal disc sample preparation method.

**Figure 5:**
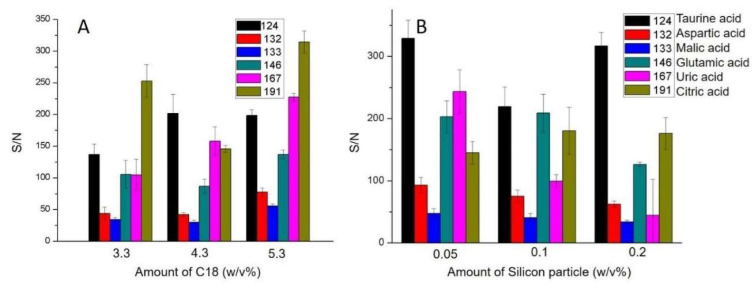
Signal-to-noise ratio of different metabolites in blood samples prepared on-chip with different densities of C18-coated silica particles (**A**) and silica nanoparticles (**B**). C18 beads are able to absorb lipids released by methanol because of hydrophobic interactions. Silica nanoparticles are able to absorb remaining proteins because of electrostatic interactions. The optimal clean-up performance was obtained by using 5.3% (*m*/*v*) C18 particles and 0.05% (*m*/*v*) bare silica particles.

Figure 5A shows that the sample prepared with 5.3% (*m*/*v*) C18 yielded higher S/N than samples prepared with less C18, so this loading of C18 beads was utilized in subsequent studies. Figure 5B compares the S/N values of the samples prepared with different doses of silica particles. Using 0.05% bare silica particles gave higher S/N values than other amounts. An excess of silica nanoparticles is likely to adsorb analytes, but the small amount that is required is sufficient to remove trace remaining proteins. The MALDI-MS results of the prepared sample without particle cleaning suffered from extremely high chemical noise due to the existence of biomolecules [19]. To summarize, densities of 5.3% (*m*/*v*) C18 coated silica particles and 0.05% (*m*/*v*) bare silica particles achieved the most effective sample clean-up.

### 3.3. Centrifugal Disc Preparation Coupled with LC-MS

**Figure 6:**
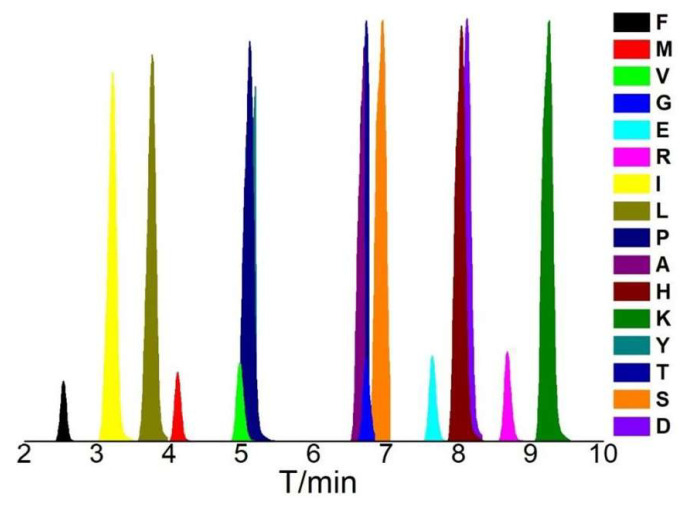
LC-MS analysis of 16 underivatized amino acid (F: Phenylalanine, L: Leucine, I: Isoleucine, M: Methionine, Y: Tyrosine, V: Valine, P: Proline, A: Alanine, T: Threonine, G: Glycine, S: Serine, E: Glutamic acid, D: Aspartic acid, H: Histidine, R: Arginine, K: Lysine) in the disc-processed blood sample using a HILIC column. Peaks are exhibited by different colours, and the corresponding amino acids for each peak are listed on the right as one-letter abbreviations.

To verify the coupling of the disc-processed samples, two different kinds of MS detection methods were utilized. HILIC LC-MS has become a standard method, and was employed to demonstrate the coverage of 16 underivatized amino acids (AAs) in a blood sample, following Agilent’s protocol for these 16 AAs [40]. Initial studies were performed on the 16 AAs in buffer using the HILIC LC-MS method following preparation with the disc protocol. As Figure 6 displays, AAs in the disc-processed blood sample were successfully analysed by LC/MS in positive mode with the combination of HILIC; the retention time and the corresponding *m*/*z* of each analyte are listed in Table 2. We abandoned showing the Y-axis in Figure 6, as the peaks were adjusted to the appropriate scale to appear in the same figure. Otherwise, the high peaks of some analytes make small elution peaks of other analytes relatively non-evident. The original chromatogram is demonstrated in Figure S3. A standard mix of amino acids was prepared according to the Agilent protocol. Similar ion counts were observed for the blood sample and the standard mix, indicating that major ion suppression problems present in a whole-blood or plasma sample were removed by the disc preparation procedure. Other than leucine/isoleucine, no two *m*/*z* values obtained from extracted ion chromatograms were observed to be the same. Consequently, the HILIC-MS study also verified that *m*/*z* peaks in MALDI-MS data could be correctly matched to the expected target amino acids. Thus, even though MALDI-MS/MS fragment data were not obtained, we could still confirm that there was no other chemical with a similar mass sharing one *m*/*z* peak when using MALDI-MS for quantification, other than leucine/isoleucine.

### 3.4. Fast Estimation of Target Molecules by MALDI-MS

Using the perfluoro-coated silicon GLAD films for MALDI-MS allows for the study of a large range of amino acids, small organic acids, and other low-molecular-weight metabolites. In contrast, HILIC LC-MS is suited for analysing small polar molecules like amino acids, but eluting strong polarity acids with amino acids under the same setting and column is still challenging. The base range, detection limits, and capabilities of the MALDI-MS method once sample clean-up has been completed have previously been demonstrated [19]. Herein, we have demonstrated the rapid analysis of an amino acid and one acid with easily available isotope internal standards by MALDI-MS, utilizing the integrated, automated disc procedure described here to perform single-point calibration assays. The isotope standard was added before the blood sample was processed by the microfluidic device, so any matrix effects or impact of selective adsorption were substantially compensated. We have previously shown that the MALDI-MS method gives quantitative results in agreement with the more complex, multi-step standard addition calibration curve-based HILIC-MS method [19]. Therefore, the MALDI-MS quantitative results were not compared with results from other detection approaches again. To align with the simplicity of the centrifugal microfluidic device, a one-point isotope standard addition method was adopted for rapid MALDI-MS semi-quantification. This means that the concentration results were read by comparing the ion counts of the isotope standard (Is) and the corresponding analyte (Ix). Table 3 shows that the concentration results for glutamic acid and citric acid in a fresh blood sample agreed with concentration data from the human metabolite database (HMDB) for healthy adults.

## 4. Discussion

Herein, we have demonstrated a centrifugal microfluidic device that can effectively automate the end-to-end sample preparation and clean-up of a whole blood sample, allowing quick and reliable sample processing for downstream small molecule analysis with mass spectroscopy methods. The device, which can be operated on a standard laboratory centrifuge, shows a wide range of coverage of amino acids when coupled with the LC-MS. The convenient, nanoporous, perfluoro-labeled, desalting GLAD thin films provided sensitive, quantitative analysis. MALDI-MS measurements also extended the small ionic molecule analysis to the organic acids analysis. The successful coupling with both LC-MS and MALDI-MS showed significant potential for this platform in terms of sample handling and automation for different sets of applications. In the future, we plan to further demonstrate the capabilities of the platform with clinical sample studies to quantify specific small-molecule-related disorders and diseases. The platform is well suited for the batch clinical analysis of samples with easy fixation to normal benchtop centrifuge, which provides potential usefulness as routine research and clinical analysis assay. Additionally, given the ease of instrumentation needed, we expect the applications of such devices in sample collection for samples that may contain time- and storage-sensitive biomarkers, thus requiring immediate sample processing, which can directly impact the sensitivity and selectivity of the final spectroscopy analysis.

## Figures and Tables

**Table 1:** List of the function of each chamber and the releasing frequency of each subsequent valve.

Chamber	Function	Release Rotational Speed/rpm
A	Blood cell separation	2400
B	Serum metering	Once the DF tab is dampened
C	Protein precipitation by MeOH	3000
D	Preload MeOH	NA
E	C18/Silica particle clean up and the sample drying	NA
F	Facilitate pneumatic mixing	NA

**Table 2:** The retention times of amino acid elutes by the HILIC column.

AAs	tR/min	*m*/*z*	AAs	tR/min	*m*/*z*
F	2.62	166.1	T	6.61	120.0
L	3.70	132.1	G	6.68	76.0
I	3.21	132.1	S	6.87	106.1
M	4.28	150.0	E	7.62	148.1
Y	5.14	182.1	D	8.08	134.0
V	5.08	118.1	H	7.99	156.1
P	5.10	116.1	R	8.70	175.1
A	6.54	90.1	K	9.15	147.1

**Table 3:** MALDI-MS quantitative results for glutamic acid and citric acid in the human blood sample.

Analyte	Internal Standard	[s]/μM	Ix/Is	[x]/μM	C in HMDB/μM
l-Glutamic acid	l-Glutamic acid-^15^N	50.01	0.82	41.01	65.2 ± 48.72 [41]
Citric acid	Citric acid-1,5-^13^C_2_	100.04	1.26	126.05	80.2 ± 44.9 [23]

## Data Availability

Data are contained within the article.

## Supplementary Materials

The following supporting information can be downloaded at: https://www.mdpi.com/article/10.3390/mi14122257/s1, Video S1: On-disc blood sample preparation; Figure S1: The original photo of the assembled disc and disc design with annotation(mm) in AutoCAD; Figure S2: MALDI-MS spectrum of the manually processed blood sample. Figure S3: HILIC-MS chromatogram for the disc-processed blood sample.
