# Supplementary material for: An Automated Centrifugal Microfluidic Platform for Efficient Multistep Blood Sample Preparation and Clean-Up towards Small Ion-Molecule Analysis
Source: Micromachines (Basel). 2023 Dec 18;14(12):2257. doi: 10.3390/mi14122257 (PMC10745919; doi:10.3390/mi14122257)
Supplement: Supplementary file 1 [file micromachines-14-02257-s001.zip › micromachines-2771865-supplementary.pdf]

Supplementary Information

An Automated Centrifugal Microfluidic Platform for Efficient Multistep Blood Sample Preparation and Clean-Up toward Small Ion-Molecule Analysis

Yuting Hou [1,*], Rohit Mishra [2,3], Yufeng Zhao [1,4,5], Jens Ducrée [3], D. Jed Harrison [1,2]

1 Department of Chemistry, University of Alberta, Edmonton, AB T6G2G2, Canada; yufeng@ualberta.ca (Y.Z.); jed.harrison@ualberta.ca (D.J.H.)

2 FPC@DCU—Fraunhofer Project Centre for Embedded Bioanalytical Systems at Dublin City University, Dublin 9, Ireland; mishrar@tcd.ie

3 School of Physical Sciences, Dublin City University, Dublin 9, Ireland; jens.ducree@dcu.ie

4 Centre for Research and Applications in Fluidic Technologies, National Research Council Canada, Toronto, ON M5S3G8, Canada

5 Leslie Dan Faculty of Pharmacy, University of Toronto, Toronto, ON M5S3M2, Canada

* Correspondence: yuting.hou@ualberta.ca

Video S1: On- disc blood sample preparation. The function of each chamber and burst frequencies of the affiliated DF valves are listed in the table 1. In detail, blood cell separation happens in chamber A under constant rate spinning for five minutes. The plasma is transferred to the metering chamber B. The big chamber in B is designed to hold and meter 50 microliter plasma, and the surplus will go to the small side chamber. When the DF tab in the side chamber is damped and destroyed, the metered 50 microliter plasma could be released to chamber C. In chamber C, the income plasma reacts with the preloaded methanol, which is preloaded by chamber D. The mixing is conducted with oscillation to ensure thorough mixing. Lately, the spinning speed adjusted to a constant rate to sediment precipitated proteins, which is lower than the burst rate of the affiliated DF valve. The supernatant is transferred to chamber E to incubate with preloaded silica and C18 particles to furtherly clean up proteins and lipids. The fully sealed chamber F is configured to facilitate the reaction under oscillation. Similarly, 15 minutes of high-speed constant spinning is applied to sediment particles and dry out Methanol. The wide vent slot on the top of the chamber facilitated quick air drying. In the end, the clean sample is ready to be pipetted out from tape sealed middle hole for downstream analysis.

https://drive.google.com/file/d/1ffAo-ZVwsmH-4OJg4_wzinZvf1fuu2ik/view?usp=sharing

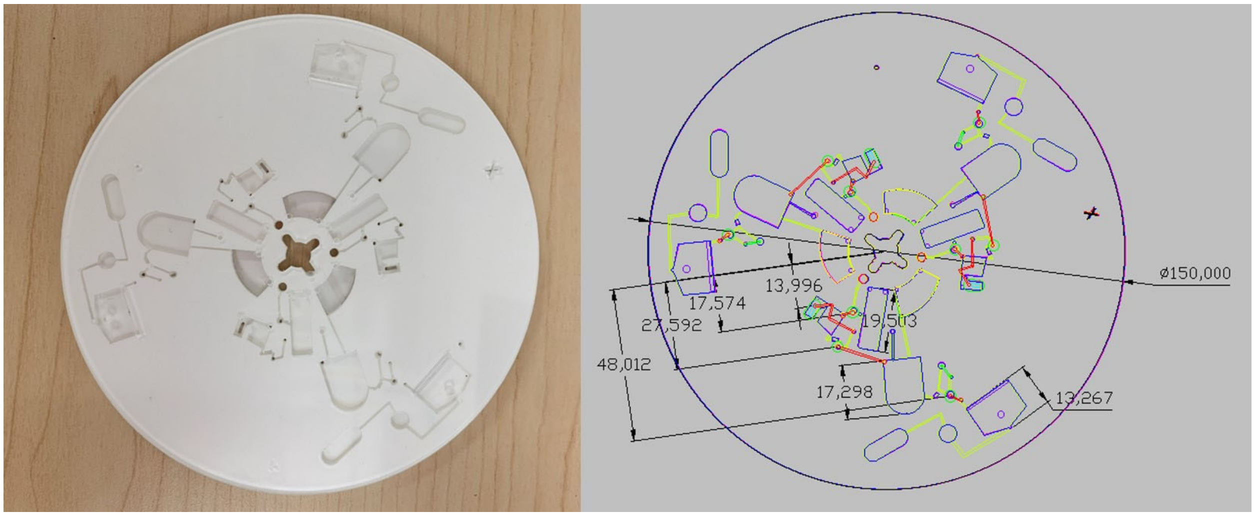

Figure S1. The original photo of the assembled disc(left) and the disc design with annotation(mm) in AutoCAD(right). Different layers are labeled with different color and stacked together. Not all annotations are indicated, only the dimension of the disc and the radial position of the DF valves and the height of the main chambers are noted.

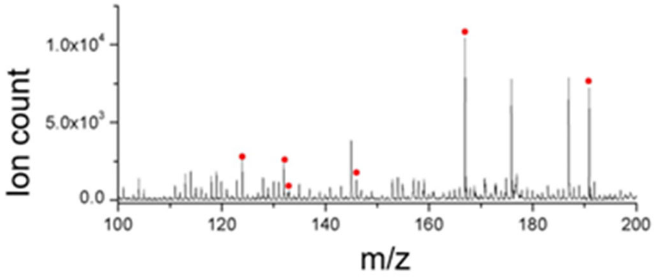

Figure S2. MALDI-MS spectrum of the manually processed blood sample.

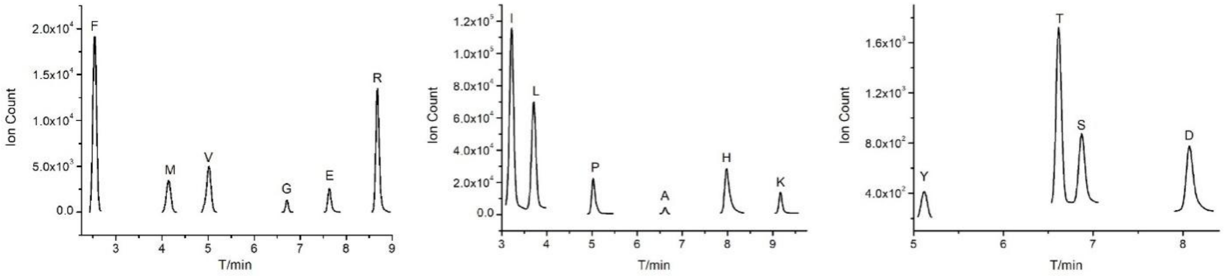

Figure S3. HILIC-MS chromatogram for the disc-processed blood sample. 16 AAs(F: Phenylalanine, L: Leucine, I: Isoleucine, M: Methionine, Y: Tyrosine, V: Valine, P: Proline, A: Alanine, T: Threonine, G: Glycine, S: Serine, E: Glutamic acid, D: Aspartic acid, H: Histidine, R: Arginine, K: Lysine) are divided into three batches to be fully separated according to the method developed by the mixture of standards.
